# Clinical significance of interleukin-1 genotype in smoking patients as a predictor of peri-implantitis: A case-control study

**DOI:** 10.4317/medoral.20655

**Published:** 2015-10-09

**Authors:** Cristina García-Delaney, Maria-Ángeles Sánchez-Garcés, Rui Figueiredo, Alba Sánchez-Torres, Cosme Gay-Escoda

**Affiliations:** 1 DDS. Master of Oral Surgery and Orofacial Implantology. School of Dentistry of the University of Barcelona, Spain; 2 MD, DDS, MS, PhD, Associate Professor of Oral Surgery. Master’s Degree Program in Oral Surgery and Implantology, School of Dentistry, University of Barcelona, Barcelona. Researcher of the IDIBELL Institute, Barcelona, Spain; 3 DDS, MS, PhD, Associate Professor of Oral Surgery. Master’s Degree Program in Oral Surgery and Implantology, School of Dentistry, University of Barcelona, Barcelona. Researcher of the IDIBELL Institute, Barcelona, Spain; 4 DDS. Fellow of the Master of Oral Surgery and Orofacial Implantology. School of Dentistry of the University of Barcelona, Spain; 5 MD, DDS, MS, PhD, Chairman and Professor of Oral and Maxillofacial Surgery, School of Dentistry, Barcelona. Director of the Master`s Degree Program in Oral Surgery and Implantology (EHFRE International University/FUCSO). Coordinator/Researcher of the IDIBELL Institute. Head of the Oral Surgery, Implantology and Maxillofacial Surgery Department of the Teknon Medical Center, Barcelona, Spain

## Abstract

**Background:**

Interleukin-1 (IL-1) is a proinflammatory cytokine that plays an important role in the pathogenesis of periodontitis, and so it might be useful to detect high-risk cases of peri-implantitis. It has been reported that IL-1 polymorphisms and smoking habit have a synergic effect, increasing the incidence of peri-implantitis. The aim of the present study was to evaluate the relationship between IL-1 gene polymorphisms and peri-implantitis in smoking patients.

**Material and Methods:**

A case-control study was performed in 27 patients with peri-implantitis and 27 patients with healthy implants. All patients included were smokers. IL-1A-C889T, IL-1B+C3953T and IL-1RN+T2018C were identified by polymerase chain reaction (PCR) amplification in order to establish a relation between these variables and the presence of peri-implantitis. A bivariate analysis was performed and odds-ratio (OR) were calculated.

**Results:**

The incidence of peri-implantitis was significantly higher in patients with previous history of periodontitis (*p*=0.024; OR=10.9). Both groups were similar regarding IL-1A-C889T, IL-1B+C3953T and IL-1RN+T2018C genotypes. No increased risk in heavy smokers with IL-1 polymorphism was found.

**Conclusions:**

IL-1 genotypes do not seem to be good predictors of peri-implantitis in the great majority of smoking patients. Furthermore, no synergic effect was found between IL-1 genotypes and heavy smokers. Patients with a previous history of periodontitis were more prone to peri-implantitis.

**Key words:**Peri-implantitis, interleukin-1 genotype positive, case-control study, smoking.

## Introduction

The treatment of partial and total edentulism with implant-supported prosthesis is highly predictable, with a low complication rate (1,2). However, peri-implant diseases are frequent (56% of the subjects and 43% of the implants) and can eventually lead to long-term failures (3). A diagnosis of peri-implantitis is made when bone loss and bleeding on probing are observed, with or without concomitant deepening of peri-implant pockets and the presence of purulent drainage (4).

Systemic conditions (diabetes), environmental factors (smoking, alcohol, poor oral hygiene), history of periodontitis, rough implant surfaces and genetic traits have been related with peri-implantitis according to previous reports (5). However, the pathogenesis, the development and the progression of this entity varies considerably depending on individual factors. For this reason, the identification of a marker that assesses the individual risk to develop peri-implantitis would be of great interest to clinicians.

Pro inflammatory cytokines such as interleukins (IL) or tumor necrosis factor α (TNF α) are biochemical mediators that control the host response to inflammation, stimulating the production of prostaglandins (associated with bone resorption) and metalloproteinases (related with collagen degradation) (6). Interleukin-1 (IL-1) might be useful to detect high-risk cases of peri-implantitis, especially because it plays an important role in the pathogenesis of periodontitis, intervening in the processes of immunity, inflammation, tissue destruction and homeostasis (7). IL-1 is a stimulator of connective tissue catabolism, enhances the migration of leukocytes into the tissues and activates fibroblasts and immune nucleated cells that produce prostaglandin E2 and metalloproteinase. IL-1 is composed by 11 genes found in section 430-kb in the DNA of the long arm of chromosome 2, in the 2q12-q21 region. These genes produce two molecules that are genetically and biochemically different, but have similar biological functions: the IL-1 alpha (IL-1A) and IL-1 beta (IL-1B) (1,2,8). Also these genes are responsible for the synthesis of the antagonist receptor IL-1 (IL-1RN) (8).

Genetic polymorphism occurs when the gene´s structure is altered. Patients who are positive for allele 2 at IL-1A -899 and IL1B +3953 loci are described as being “genotype positive”. Only a small fraction of these genetic variations are phenotypically important, since alterations in amino acid sequence are infrequent (9). Apparently, genetic polymorphisms seem to have a regulatory effect on the secretion of these IL-1A, IL1B and IL1RN, increasing or decreasing the production according to the expressed allele. When a genetic variation occurs in the IL-1 (-889 locus in the IL-1A and +3953 locus in the IL-1B) an increased level of IL-1 is expected, therefore enhancing inflammatory activity. On the other hand, when IL-1RN is involved, the level of this substance is lower allowing more molecules to join the IL-1 receptor, also causing inflammation (10).

The role of these genetic polymorphisms in the etiology and progression of peri-implant diseases is still unclear and needs further research, with several papers showing opposing results. Another interesting aspect that has been reported is that IL-1 polymorphisms and smoking habit seem to have a synergic effect, increasing the incidence of peri-implantitis (11). Therefore, it would be interesting to perform a study to determine if IL-1 polymorphism is a risk factor for peri-implantitis and to analyze the association between IL-1 genotypes and the amount of tobacco consumption.

## Material and Methods

A retrospective case-control study involving 54 caucasian smoker patients with dental implants treated in 2012 in the Oral Surgery and Implantology master degree program of the Faculty of Dentistry, University of Barcelona (Spain) was performed. Patients were enrolled into 2 different groups (ratio 1:1) according to the following criteria: Peri-implantitis (PI) group consisted of 27 patients consecutively diagnosed with peri-implantitis according to the criteria described by Koldsland *et al*. (12) (bleeding on gentle probing (BoP) of the peri-implant tissues or suppuration and bone loss > 2mm); Control group (CG) included 27 patients that attended the dental hospital with healthy implants (no BoP, bone loss < 2mm, no suppuration).

A complete periodontal examination, with probing depth (deepest value for each implant was registered), peri-implantary Mombelli plaque and bleeding index (PPI and PBI) (13) and suppuration was made in all cases. Periapical radiographies using the long-cone parallel technique were made for the measurement of the bone loss counting the number of mm without bone support. A single researcher assessed the mesial and distal aspects of each implant, and registered the highest value. A second surgeon reviewed all radiographies, in order to confirm the diagnosis. Cases with incomplete data or with doubtful diagnosis were excluded from the analysis. All patients included in the study were smokers (> 3 cigarettes/day, >5 years of habit) and healthy (ASA I or II). The functional loading time of the implants was of 18 months or higher.

Data about the surface and position of the implants (anterior or posterior), type of prosthesis (removable or fixed), history of periodontitis and tobacco consumption (light smoker: <10 cigarretes/day; heavy smoker: ≥10 cigarretes/day) was collected.

All patients were explained the nature and objectives of the study, and signed an informed consent prior to inclusion in the study. The study protocol was reviewed and approved by the institutional review board (Ethical Committee of Clinical Investigation, University of Barcelona Dental School; reference 06/2012). The Helsinki Declaration guidelines were followed throughout the study. The Strobe Statement guidelines (14) for case-control studies were also taken into consideration when designing the current research.

- Analysis of genetic polymorphisms

**Figure 1:**
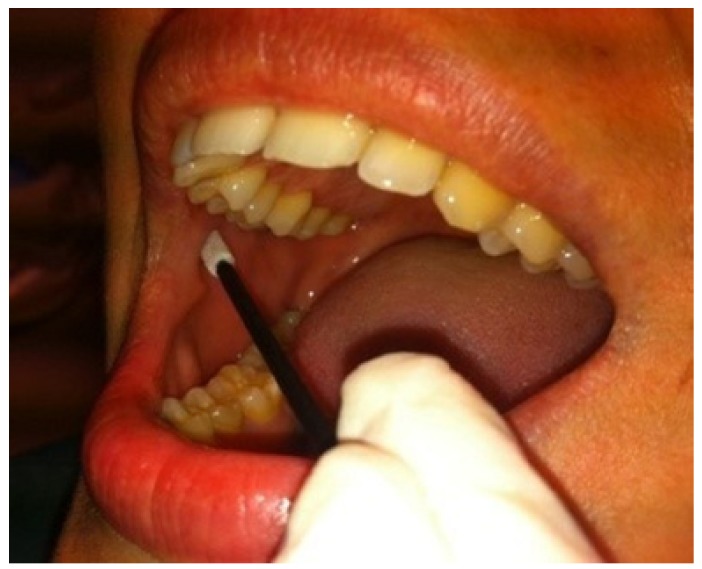
Epithelial cells were obtained with the use of a sterile cotton swab.

Epithelial cells of the oral mucosa were obtained with the use of a sterile cotton swab and the tip of the spatula was immediately placed in snap tubes (Fig. 1).

To determine genetic predisposition to peri-implantitis, the IL1 Genotype commercial PCR kit (PST, Hain Lifescience GmbH, Nehren, Germany) was used. The kit allows the detection of the following IL1-related single nucleotide polymorphisms (SNPs): IL-1A-C889T, IL-1B+C3953T and IL-1RN+T2018C. DNA was obtained from buccal swabs after extraction using QIAamp DNA Mini from QIAGEN® (Hilden, Germany). The samples were incubated for 10 min at 56ºC with AL Buffer plus proteinase K. The rest of the extraction was performed according to manufacturer instructions. PCR was performed using BIOtaq polymerase (BIOLINE®, London, U.K.) with the following program: 95ºC-5min + 10x (95ºC-30s+58ºC-2min) + 20x (95ºC-20s+72ºC-40s) + 70ºC-8min. Hybridization and detection was performed according to manufacturer instructions.

Genetic polymorphisms can arise in homozygosity or heterozygosity and their sum is considered a positive genotype.

The sample size was calculated using the software G * Power 3.0. (Heinrich-Heine-Universität, Düsseldorf, Germany). The following parameters were used: α=0.05; β=0.1; positive genotype proportion in the control group of 0.2 (according to the report by Hamdy and Ebrahem (15); and expected positive genotype proportion in the PI group of 0.4.

- Data analysis

Data were processed with the Statistical Package for the Social Sciences (SPSS version 12.0; SPSS, Chicago, Ill, USA). Normality of scale variables was explored using the Kolmogorov-Smirnov test with Lilliefors correction. Parametric and nonparametric tests (Pearson chi-square, Fisher exact tests and t-student tests) were used to compare the groups. The patients were also divided in 2 subgroups (with and without previous history of periodontitis) because this was considered an important confounding variable. An additional analysis was made in positive IL-1 genotypes patients, to determine possible synergism between heavy smokers (≥10cig/day) and IL-1 genotypes alterations in peri-implantitis. Odds ratios (OR) with 95% confidence intervals (95%CI) were calculated for each categorical variable. The level of significance was set at *p*<0.05.

## Results

The present study included 54 patients with ages between 23 and 65 years. In the PI group (n=27), the mean patient age was 54.4 years (range 40 to 66), while in the control group (n=27) the mean age was 50.6 years (range 23 to 66 years). The main clinical features of the patients, the different surfaces and position of implants and the need for guided bone regeneration before or at the time of implant placement can be observed in  Table 1. An additional analysis that divided patients into 2 groups: positive genotype (homozygosity or heterozygosity) and negative genotypes for IL-1A, IL-1B and IL-1RN, showed no significant associations between these variables and the study groups.

**Table 1:**
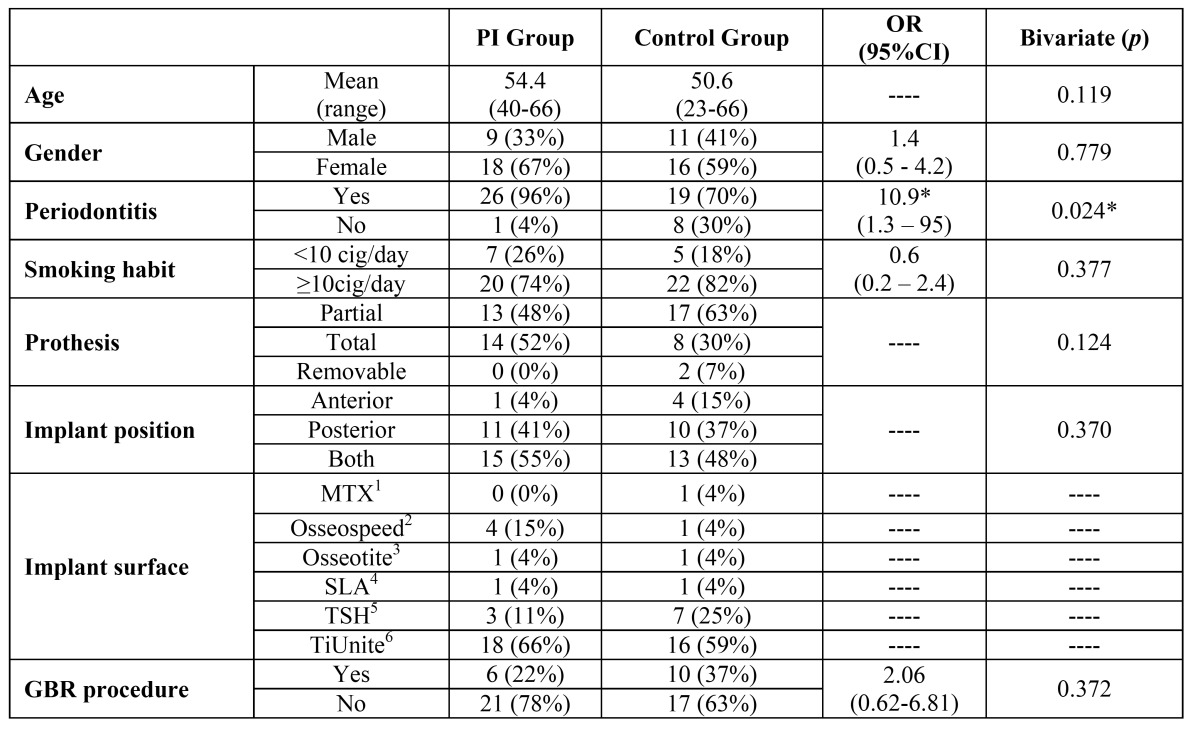
Main clinical features of the patients included in both study groups. The OR value for the variable smoking habit was calculated dividing the patients into light smokers (<10cig/day) and heavy smokers (≥10cig/day). 1Zimmer Dental, Inc, Carlsbad, CA; 2Astra Tech Implant System, Dentsply Implants, Mölndal, Sweden; 3BIOMET 3i, Palm Beach Gardens, FL, USA; 4Straumann AG, Waldenburg, Switzerland; 5Defcon, Impladent, Sentmenat, Barcelona, Spain; 6Nobel Biocare AB, Göteborg, Sweden.

Regarding the incidence of periodontitis, a statistical significant difference was found between the 2 groups, since 26 patients (96%) in the PI group and 19 patients (70%) in the control group had periodontitis. The position of the implants (anterior or posterior), the type of prosthesis (partial or removable), the amount of tobacco consumed (<10 or ≥10 cigarettes/day) and the different genotypes showed no statistically significant association with the development of peri-implantitis ( Table 1 and  Table 2).

**Table 2:**
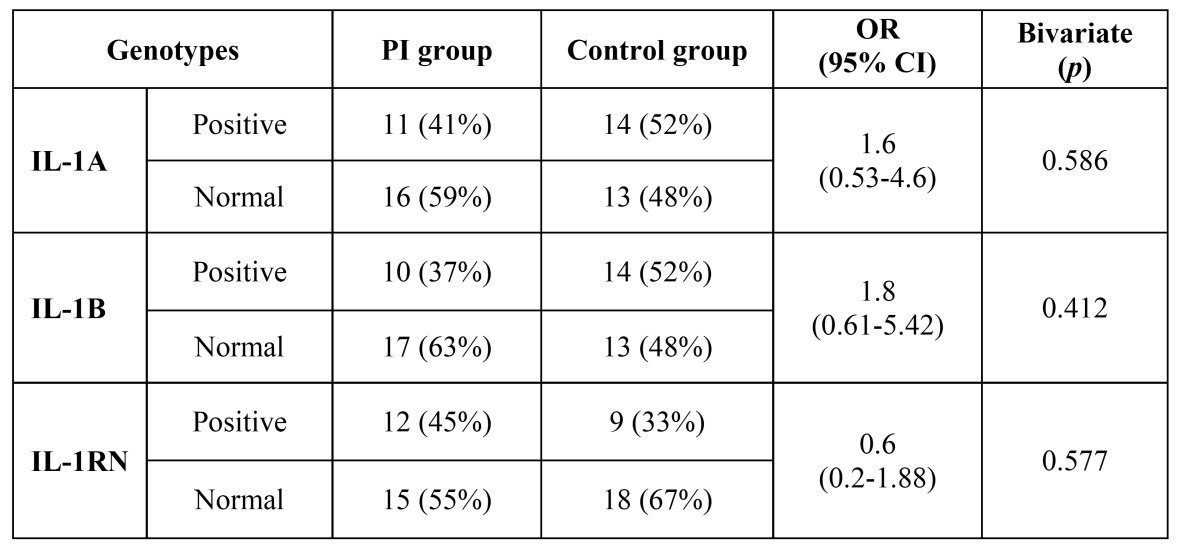
Distribution IL-1A, IL-1B, IL-1RN genotypes in both groups.

 Table 3 shows the distribution of smoking status, IL-1A, IL-1B and IL-1RN genotypes in patients with and without periodontitis.

**Table 3:**
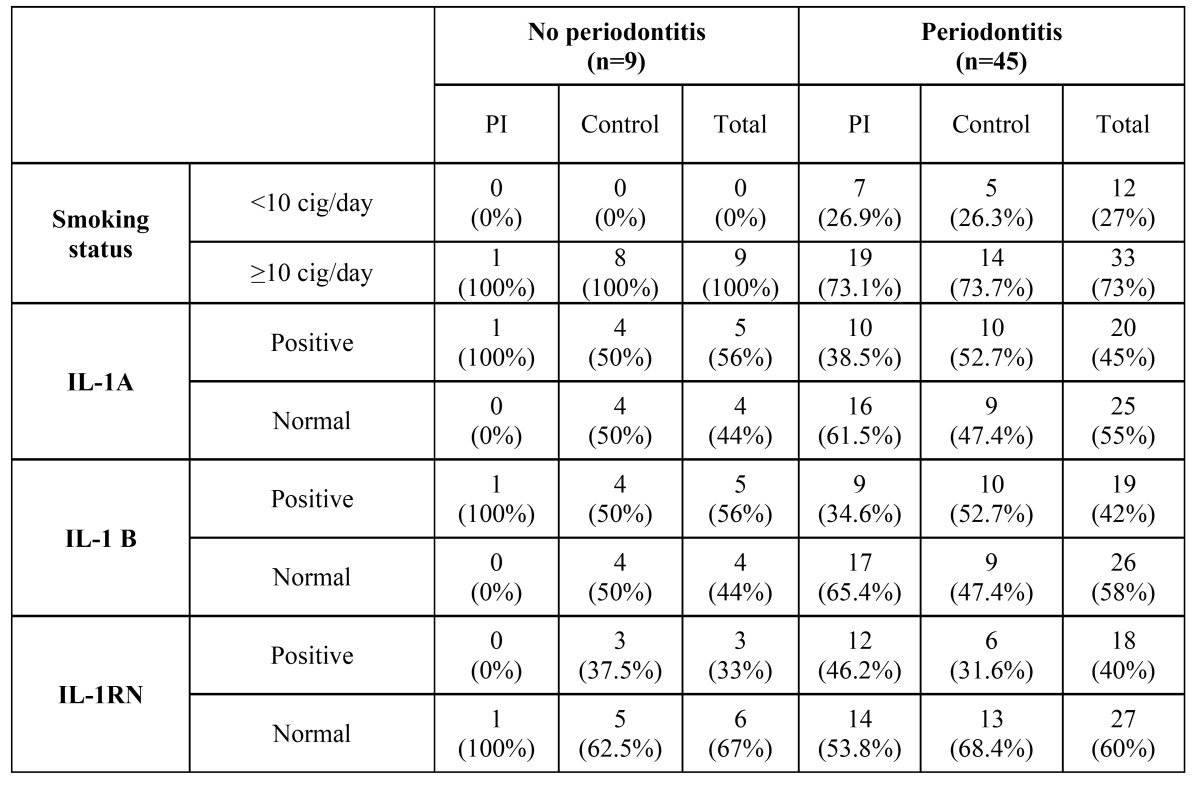
Distribution of smoking status, IL-1A, IL-1B and IL1RN genotypes in patients with and without periodontitis. Due to the small amount of patients without periodontitis in the presented sample, no statistical tests were applied.

## Discussion

In the Sixth European Workshop on Periodontology (EWP), published in 2008 (16) the only risk indicators for peri-implantitis with clear evidence were poor oral hygiene, history of periodontitis and smoking habit. On the other hand, genetic traits and implant surface were considered risk indicators with conflicting and limited evidence for an association with peri-implant diseases. The following EWP, published in 2011 (4), stated that peri-implantitis may be initiated and/or maintained by iatrogenic factors such as cement remnants, inadequate seating or overcountouring of restorations, implant mal-positioning and traumatizing of the pristine bone at the time of implant placement.

The concept that microorganisms are essential for the development of infections around dental implants is well supported in the literature. It has been suggested that periodontal pockets of teeth may act as a reservoir for microorganisms to colonize the newly inserted implants (17). Although bacteria may be considered the initiating factor of periodontal disease, other aspects like genetic factors may be extremely important. In fact, most authors agree that the host response to bacterial challenge is a critical factor, especially in the progression of the disease (18). These 2 factors (similar bacterial profile and genetic predisposition) might explain why patients with periodontitis are at great risk of developing peri-implant diseases (19). Other studies have shown this association and our report also seems to support the relation between periodontitis and peri-implantitis, with a significant OR value (OR=10.9; 95%CI=1.3 to 95) (19,20).

The identification of genetic polymorphism as a risk indicator for peri-implant infection has been investigated in a number of clinical studies with opposing results. Systematic reviews made by Dereka *et al*. (21) and Huynh-Ba *et al*. (22) stated that there is not enough evidence to support or refute an association between specific genetic polymorphism and dental implant failure respect to peri-implantitis. Therefore, systematic genetic testing to assess the risk of peri-implantitis cannot be recommended as a standard of care at this time. Lachmann *et al*. (23) seem to support this statement, as they failed to find an association between the IL-1 genotype and peri-implantitis. On the other hand, a recent meta-analysis concluded there was evidence of genetic effect (composite genotype IL-1A and IL-1B) on risk for implant failure and peri-implantitis (2). The results are different from ours probably because our study only included smoker patients, whereas the previous meta-analysis had a more broad inclusion criteria regarding this variable. In our opinion, this stresses the need to perform additional research to evaluate if the influence of positive genotype varies depending on the presence coexisting risk factors. Hamdy and Ebrahem (15), in a case-control study including 50 nonsmoking patients, found a statistically significant difference between the groups regarding IL-1 genotype detection. This reinforces the idea that genetic testing might be useful to detect high-risk cases but only in particular situations. Probably, genotype detection might be especially relevant in cases without important risk factors (non-smoking patients with good oral hygiene and without previous history of periodontitis). The lack of association between IL-1 positive genotypes and peri-implantitis in our study could be explained by the high prevalence of smokers (100%) and periodontitis (83%). According to Hamdy and Ebrahem (15), the treatment of peri-implantitis could have a worse prognosis in IL-1 positive genotype patients. The clinical relevance of detecting high-risk cases resides in the introduction of a more strict maintenance program for patients with implant-supported prosthesis. Tobacco has been reported as a risk factor for implant failure and bone loss around implants (24). A systematic review and a study made in our department also showed a significantly higher risk of developing biologic complications among smokers compared with non-smokers (25,26). In a report by Rinke *et al*. (27) the data of 89 patients were collected in a practice-based cross-sectional study. The patient-related global prevalence rate of peri-implantitis was 11.2%. However, when patients had risk factors (smoking habit and periodontal history) the prevalence increased dramatically (53.3%). Some authors (28,29) have suggested that smoking patients with IL-1 gene polymorphism might have an increased risk for peri-implant bone loss, since a synergic effect is expected. In these reports, a clear association between heavy smokers with a positive IL-1 genotype and implant complications (loss of implant, or peri-implantitis) was found. For this reason, the patients of our sample were analyzed taking into consideration the amount of cigarettes consumed in a day. However, we failed to find an increased risk of peri-implantitis in heavy smoking patients with positive IL-1 gene polymorphism.

## Conclusions

Patients with a history of periodontitis are more prone to peri-implantitis. According to the results of the present study, IL-1 genotypes do not seem to be good parameters to assess peri-implantitis predisposition in the great majority of smoking patients. Furthermore, no increased risk of peri-implantitis was found in heavy smokers with IL-1 positive genotypes.
